# Liver Trauma: Until When We Have to Delay Surgery? A Review

**DOI:** 10.3390/life12050694

**Published:** 2022-05-06

**Authors:** Inés Cañas García, Julio Santoyo Villalba, Domenico Iovino, Caterina Franchi, Valentina Iori, Giuseppe Pettinato, Davide Inversini, Francesco Amico, Giuseppe Ietto

**Affiliations:** 1 General and Digestive Surgery, Hospital Clínico San Cecilio of Granada, 18002 Granada, Spain; inescanasgarcia@gmail.com; 2 General and Digestive Surgery, Hospital Virgen de Las Nieves of Granada, 18002 Granada, Spain; jsantoyovil@gmail.com; 3 General, Emergency and Transplant Surgery Department, ASST-Settelaghi and University of Insubria, 21100 Varese, Italy; davide.inversini@gmail.com (D.I.); franchicaterina88@gmail.com (C.F.); valentina.iori@gmail.com (V.I.); domenico.iovino@asst-settelaghi.it (D.I.); 4 Department of Medicine, Beth Israel Deaconess Medical Center, Harvard Medical School, Boston, MA 02115, USA; gpettina@bidmc.harvard.edu; 5 Trauma Service, Department of Surgery, University of Newcastle, Newcastle 2308, Australia; francesco.amico@hotmail.com

**Keywords:** liver trauma, DAMPs (damage-associated molecular patterns), NETs (neutrophil extracellular traps), SIRS (systemic inflammatory response syndrome), DCS (damage control surgery), nonoperative management, liver regeneration

## Abstract

Liver involvement after abdominal blunt trauma must be expected, and in up to 30% of cases, spleen, kidney, and pancreas injuries may coexist. Whenever hemodynamics conditions do not contraindicate the overcoming of the ancient dogma according to which exploratory laparotomy should be performed after every major abdominal trauma, a CT scan has to clarify the liver lesions so as to determine the optimal management strategy. Except for complete vascular avulsion, no liver trauma grade precludes nonoperative management. Every attempt to treat the injured liver by avoiding a strong surgical approach may be considered. Each time, a nonoperative management (NOM) consisting of a basic “wait and see” attitude combined with systemic support and blood replacement are inadequate. Embolization should be considered to stop the bleeding. Percutaneous drainage of collections, endoscopic retrograde cholangiopancreatography (ERCP) with papilla sphincterotomy or stent placement and percutaneous transhepatic biliary drainage (PTBD) may avoid, or at least delay, surgical reconstruction or resection until systemic and hepatic inflammatory remodeling are resolved. The pathophysiological principle sustaining these leanings is based on the opportunity to limit the further release of cell debris fragments acting as damage-associated molecular patterns (DAMPs) and the following stress response associated with the consequent immune suppression after trauma. The main goal will be a faster recovery combined with limited cell death of the liver through the ischemic events that may directly follow the trauma, exacerbated by hemostatic procedures and surgery, in order to reduce the gross distortion of a regenerated liver.

## 1. Introduction

The liver is one of the solid organs most frequently affected in abdominal trauma. We can find associated lesions in up to 30% of cases, especially of the spleen, kidney, and pancreas [1,2]. Advances in diagnostic and therapeutic techniques, multidisciplinary approaches in recent years, together with the emergence of advanced endovascular techniques, have increased the probability of non-operative management (NOM) in selected patients and experienced centers in trauma surgery, leaving apart the classic dogma abdominal trauma—exploratory laparotomy [3].

A detailed and systematized physical examination is a priority and is mandatory in the decision-making algorithm at emergency department admission. Complementary imaging tests will depend on the stability of the patient: if systolic blood pressure <90 mm Hg (despite adequate fluid therapy, blood products or vasopressors), as well as in case of an altered level of consciousness, urgent exploratory laparotomy should be evaluated [1,4,5]. If the patient shows hemodynamic stability or good response to initial volume resuscitation, an abdominal computed tomography (CT) scan with intravenous contrast should be performed. The CT scan is considered the *gold standard* for the diagnosis of post-traumatic abdominal injuries, although the echo-fast technique can be useful, with less sensitivity and specificity [1,2,3,4,5,6].

The liver trauma is classified according to the American Association for the Surgery of Trauma (AAST) into six grades of severity.

The CT anatomical characterization of hepatic injuries has a vital role to define the optimal treatment strategy. Furthermore, the hemodynamic stability, the association of other visceral lesions and the initial response to volume replacement will determine the decision of NOM. A CT scan should be carried out whenever conservative management is chosen [6,7].

Following the recommendations provided at the 2020 World Society of Emergency Surgery (WSES) consensus conference, NOM should be the treatment of choice in hemodynamically stable patients with grade injuries I-V of the AAST classification when there are not any associated visceral lesions which need urgent surgery [5,7]. Additionally, the availability of hospital center resources must be taken into account: intensive care unit, endovascular techniques, blood products, as well as a team of expert surgeons [8]. In stable patients presenting with blushing or active bleeding on the CT scan, angioembolization should be considered as the gold standard treatment. The procedure can be repeated if hemodynamic stability persists and the bleeding has not been solved. Diagnostic laparoscopy can be valued as an option in the context of NOM if, in close follow-up, there are reasonable doubts about the existence of possible associated visceral injuries [7,8,9,10].

In conclusion, the degree of liver injury is an essential factor to take into account in the decision algorithm and even if the patient is hemodynamically stable, the indication of conservative treatment and the admission to specialized centers for close monitoring with frequent clinical exams by expert abdominal trauma surgeons could be advisable [1,11,12,13].

## 2. Nonoperative Management: Wait and See

The management of hepatic trauma has evolved significantly over the past few decades. In the past, the decision between nonoperative and operative management depended primarily on the free volume of blood. Past guidelines on hepatic trauma suggested a tolerable volume of approximately 500 mL to manage it with nonoperative techniques. Considering the current WSES guidelines, the management of hepatic injuries is based primarily on the status of hemodynamics of the patients, plus other associated injuries, rather than the radiological severity of the injuries [1]. In hemodynamically stable patients and even in borderline or transient responder patients with no other indication for laparotomy, NOM could be considered as standard of care in both blunt and penetrating liver trauma. This strategy needs a multidisciplinary approach and can only be used in trauma centers. Mild and moderate lesions are the most common (80% to 90% of all cases); grade VI lesions are often incompatible with survival. In the current medical literature, most patients treated with NOM have low-grade liver lesions. The safety of NOM in high-grade liver injuries, AAST grade IV and V, remains a matter of debate because of the high incidence of hepatic and extra-abdominal complications. During NOM, a serial clinical evaluation and continuous laboratory testing are necessary to detect a change in the patient’s clinical condition; radiologic follow-up is performed only with ultrasound of the abdomen. If there is clinical suspicion of NOM failure, or in case of complications, the CT scan has to be repeated. Among major risks related to NOM, especially in penetrating trauma, we can count the presence of intra-abdominal unacknowledged lesions. The laparoscopic interval exploration could be considered as part of non-operative liver injury management, playing an important diagnostic role in the therapy planning. On the one hand, NOM is a standard of care in stable patients even if the liver injury is severe (AAST IV) and even in cases of CT-detected venous blush, but on the other hand it is absolutely contraindicated in the following scenarios: if intra or retroperitoneal free air is detected on CT; in presence of intraperitoneal free fluid in the absence of other solid organ injury; with localized thickening of the bowel wall; in case of penetrating trauma if the bullet tract is near the hematoma created adjacent a hollow viscus and in high intensity trauma [14,15,16].

## 3. Failure of NOM: Acute and Subacute Consequences of Liver Trauma

The introduction of NOM and the improved operative and perioperative care have significantly decreased mortality. Nevertheless, the rate of liver-related complications in patients with high-grade liver injury is 12–14% and could be predicted by the degree of liver injury and the volume of packed red blood cells transfused within the first 24 h after injury [4,9,17,18].

Late bleeding, bleeding after embolization, biliary complications (bile leakage, biloma, hemobilia, biliary peritonitis, biliary fistula), hepatic abscess, abdominal compartment syndrome, ischemic necrosis of the liver and the gallbladder are some of the most common complications [19,20,21]. 

Advances in endovascular techniques and therapeutic digestive endoscopies (ERCP) give furtherance to the multidisciplinary management of these complications, allowing further progress in NOM [22]. 

In relation to delayed bleeding or hepatic artery pseudoaneurysm, angioembolization should be considered. Regarding pseudoaneurysm, although rare, <1%, the risk of rupture carries high morbidity and mortality, for which prophylactic treatment is recommended after diagnosis [1,2]. 

Post-traumatic biliary complications occur with an incidence of 2.8–30% and are treated first with percutaneous and endoscopic procedures although most traumatic bilomas regress spontaneously [23]. An ERCP with endoscopic stent placement could be associated with percutaneous drainage as a therapeutic strategy [1,23,24]. 

Percutaneous drainage of abscesses and intrahepatic bilomas should be considered as the first option in stable patients with high serum level of inflammation markers, abdominal pain or fever [22,25].

In addition, the combination of ERCP techniques and percutaneous biloma drainage should be considered as an initial option in the treatment of late biliary fistulas [1,2,22,25].

A possible indication for surgery could be the necrosis of a hepatic segment.

To conclude, minimally invasive interventions are the first choice of treatment for later complications in stable patients [1,23,24,26].

## 4. Embolization

According to WSES guidelines, in patients with stable hemodynamics and active arterial redness, angioembolization (AE) should be considered the first-line intervention and, in selected centers, represents an extension of NOM. In the event of an ineffective procedure, serial clinical, laboratory, and radiologic evaluation must be performed, and if necessary, AE can be safely repeated [1,20]. Surgically inaccessible regions deep within the hepatic parenchyma make selective embolization after hepatic packing a viable supplemental option [27]. Active extravasation of CT contrast is suggestive of potentially life-threatening bleeding. Such radiological findings or clinical signs of active bleeding require arteriography and embolization. Embolization agents are divided into two categories: temporary (Gelfoam, autologous clot) and permanent (coils or microcoils, particles, occlusion devices, glue, and onyx). Coils, microcoils, and Gelfoam cubes are the preferred embolic materials used in liver trauma. The embolization is performed on both sides of the injured vessel. If the bleeding area cannot be reached with a selective modality, embolization is practiced in the proximal vessels with occlusion devices or larger particles. AE is the safest and most successful method for controlling of active hepatic arterial bleeding and is associated with a low rate of transfusions and the need for surgical intervention. Although it is not free of complication, AE appears to improve mortality rates for severe liver trauma [28,29].

## 5. Percutaneous Drainage of Fluid Collection

Intra-abdominal fluid collections such as abscess, biloma, lymphocele, hematoma, and seroma following hepatic resection and trauma are common complications. Abscesses are associated with significant morbidity and mortality and require early and effective treatment [30,31]. Ultrasound-guided drainage is safe and advantageous if a fluid collection can be easily visualized. The procedure has the benefit of avoiding radiation exposure and low cost, although the operator needs to be highly experienced. In addition to antibiotic therapy, CT-guided percutaneous drainage represents an alternative to surgery and is currently the standard of care. Compared with surgical drainage, it has the advantage of being less invasive and, if needed, could be repeated. The early use of percutaneous CT-guided drainage for the management is widely accepted as a mainstay of treatment [32,33,34].

## 6. Endoscopic Retrograde Cholagiopancreatography (ERCP): Papilla Sphincterotomy and Stenting

Biliary leakage secondary to blunt or penetrating hepatic trauma remains a challenging concern. The study by Lubezky et al. represents the largest reported series of patients with biliary leakage following hepatic trauma and shows a successful resolution of the leak in 90–100% of the cases with the use of ERCP and placement of a trans papillary plastic stent. Stenting reduces the pressure gradient between the bile duct and duodenum, abolishing the role of Oddi’s sphincter. Bile is drained into the duodenum, allowing the interrupted duct to recover spontaneously. In conclusion, the resolution of the bile leak can be achieved by sphincterotomy alone, stent placement alone, or a combination of the two interventions [35,36].

## 7. Percutaneous Transhepatic Biliary Drainage (PTBD)

The need for prompt control of hepatic inflow and outflow structures is a crucial concern in trauma surgery in contrast to elective settings. In the case of trauma, the primary goal is to control the hemorrhage; the patency of the bile duct is still important but represents a secondary objective, especially in patients with hemodynamic instability. The reported incidence of postoperative biliary complications increases with the severity of liver injury [37,38]. Non-invasive approaches are recommended as first options in such complications. PTBD is superior for proximal bile duct stenosis and common bile duct or aberrant right hepatic bile duct injuries compared with ERCP. PTBD is considered the first choice of treatment in the following cases: failure of ERCP; if a complete ductal ligation or transection occurred; if the stenosis is located in a proximal bile duct and in case of immediate need for decompression. In case of trauma, the priority is draining the bile duct, which should be punctured using a 21 G or smaller needle with ultrasound guidance and, at a later time, pass the stenosis. The stenosis should not be forced through rough and sudden maneuvers in fragile livers, to avoid the risk of complications [39].

## 8. Laparoscopy

The laparoscopic approach has a vital role in the management of hepatic trauma, with the advantages of a clear vision of the whole abdominal cavity and high safety [40]. Interval laparoscopy is an effective strategy to evaluate the lesion and its progression while minimizing the damage and invasiveness of the surgical procedure [1]. It could be used as a bridge to other procedures. In addition, laparoscopy should be considered an alternative to percutaneous radiological drainage and ERCP in stable patients with blunt liver trauma and hemorrhagic-bilious ascites, while avoiding the morbidity of open surgery [41].

## 9. Laparotomy

Hemodynamically unstable patients should undergo operative management with an exploratory laparotomy. Approximately 40% of all penetrating abdominal trauma requiring urgent laparotomy due to uncontrollable bleeding from the liver. It is critically important to provide intensive intraoperative resuscitation with the establishment of a massive transfusion protocol to maintain organ perfusion and to reverse derangements induced by the trauma itself [26]. The primary goal is to control hemorrhage and bile loss by performing damage control surgery. The exploratory laparotomy allows the complete exposure of the abdominal cavity. During the procedure, it is crucial to store and measure the blood loss and eventually proceed to auto-transfusion. In case of minor bleeding, bimanual compression of the hepatic parenchyma may be sufficient. This maneuver can be complemented by perihepatic packing with swabs. Topical hemostatic agents, coagulation with argon, bipolar devices could be used to stop the bleeding. The surgeon could even decide to perform a simple suture of the injured parenchyma with or without omental packing. If bleeding persists, it may be an arterial source and it is confirmed by slowing of the bleeding by clamping the hepatic pedicle with a Pringle maneuver [42,43]. If hemorrhage endures, the surgeon must contemplate the presence of an anomalous hepatic artery. In presence of lesion to the proper hepatic artery, attempts should be made to control and fix it and if ineffective, selective ligation should be considered. Cholecystectomy should be performed in case of ligation of the right or common hepatic artery to prevent gallbladder necrosis [44]. Hepatic necrosis, development of abscesses and biloma are the most frequent risks recorded in case of hepatic artery ligation; therefore, in lobar or segmental/subsegmental injuries of a portal vein branch, the surgeon should choose between liver packing or resection instead of hepatic artery ligation. Ligation of the main branch of the portal vein should not be considered on account of the risk of hepatic necrosis and bowel edema [26]. Retrohepatic vena cava injury has a well-known high mortality (65–100%). In this case, there are three viable options: packing, direct synthesis, and lobar resection [9,45]. In the current literature, a lot of vascular exclusion techniques with shunting procedures have been reported, and the veno-venous bypass and the use of fenestrated stent grafts are the most widely used [1,9,44]. By the application of a chest tube into the inferior vena cava, the bypass of the retrohepatic cava blood is achieved through the right atrium; this technique is called atrio-caval shunt. Mainly, the unstable patients do not tolerate the procedure of complete liver vascular exclusion [26]. Another important technique that should be consider in treatment of hemodynamically unstable patients is REBOA (resuscitative endovascular balloon occlusion of the aorta); this could be performed in case of continuous bleeding by an active source when the other damage control procedures are ended [46,47].

## 10. Resection: When and What

Major hepatic resections should only be considered in later operations when large areas of necrotic parenchyma are involved and only performed by skilled surgeons [1,48]. Non-anatomic resections refer to the removal of devitalized parenchyma using the line of injury as resection borders rather than standard anatomic planes. Such procedure should be made to limit the extent of hepatic resection, with the advantage of a shorter operative time [15,49]. It represents a safer and easier alternative than anatomic resections [1]. However, is favorable to avoid it in treatment of unstable patients and during damage control procedures. Extensive dissection through the uninjured parenchyma should not be performed [50]. In the case of anatomic resection, the parenchyma is removed along anatomic planes after the identification of inflow and outflow vessels. Extensive anatomic resections are characterized by longer operative times, higher mortality, and morbidity rates. Such resections are rarely performed, only in 2–4% of cases of major liver trauma [15,17,50].

## 11. Transplantation

Liver transplantation represents a lifesaving procedure in severe liver trauma when all other treatments have failed. There have been 19 cases in the literature of total hepatectomy and liver transplantation performed in trauma patients [1,51,52,53]. Ringe and Pichlmayr reported the largest series of 8 patients. Liver transplantation is the only therapeutic option for progressive acute liver failure, in major trauma in uncontrollable bleeding, severe grade IV-V injury, irreversible acute liver failure and life-threatening post-reperfusion injury. Different surgical options exist once the decision to proceed with transplantation is achieved. The first is a two-staged procedure with total hepatectomy and portocaval shunt followed by transplantation. The second option is to continue supportive management while the patient waits for a suitable donor transplant with subsequent standard liver transplantation [54,55].

## 12. Pathophysiology of Liver Trauma: Local and Systemic Imbalance

Understanding the pathological mechanisms that develop after liver trauma is critically important, both in determining patient’s outcome and in making choices in different therapeutic strategies.

To reduce the stress response and the following immune suppression [56] after trauma, it is quite clear that it is fundamental to minimize infection, not only using an adequate antibiotics prophylaxis but especially through the adequate nutritional support to preserve the homeostasis. To this end, reducing every possible additional trauma due to any operative post-injury management (as would be necessary for surgery) plays a pivotal role. The severity of the initial damage and the magnitude of the immune system’s reaction are directly related to the wide spectrum of symptoms that develop after trauma and in all critical diseases.

The point of fact, after every traumatic injury, almost instantaneously, or at least very rapidly, is that a large amount of cells die, resulting in a freeing of cellular fragments and damage associated molecular patterns (DAMPs) as mtDNA and histones. This leads to the consequences presented in the so-called “Danger model”.

Matzinger’s “Danger model”, presented in 2002, is built on the concept that the immune surveillance does not discriminate among self and non-self, but between damaging and non-damaging events [57,58,59], through the identification of warning alerts from pathogens or damaged tissues and cells.

As proposed by Janeway in 1989 [60], antigen-presenting cells (APCs) are in a dormant state until triggered via pattern recognition receptors (PRRs). PRRs are located on the membrane of APCs known as toll-like receptors (TLRs) [60,61], and in the cytoplasm called the nucleotide-binding oligomerization domain (NOD)-like receptors (NLRs) [62]. They detect pathogen-associated molecular patterns (PAMPs) on bacteria and fungi and DAMPs, launching the immune reaction.

Simultaneously, such components ligate to NOD, which, along with Leucine Rich Repeat (LRR) and pyrin domains containing protein 3 (NLRP3), form the inflammasome subunit. As a result of the triggering of the NOD domain, NLRPs oligomerizes, leading to activation of the inflammasome. This carries to cleavage of pro IL-1β and pro IL-18 into activated forms through the action of caspase 1 and gasdermin D-mediated pyroptotic cell death [63,64].

IL-1β is the mediator of lung inflammation, fever, and fibrosis. DAMPs spreading after pyroptosis amplifies the cascade and potentiates the inflammation.

Costimulatory molecules become activated on APCs which process the foreign antigens and show them to the in-transit T cells. Moreover, cytokines and PAMPs upregulate the canonical and non-canonical inflammasome components through a transcriptional adjustment [65].

Neutrophil function and neutrophil extracellular traps (NETs) are other crucial factors implicated in the earliest events of immune activation.

Although NETs play important roles by trapping pathogens, their extensive formation with increased amounts of extracellular DNA may contribute to the perpetuation of inflammation and tissue damage [66,67,68,69,70,71,72,73,74,75]. Contemporary studies suggested their role in the activation of platelets, causing coagulative disorders, thrombosis, injuries of endothelial cells and organ damage [74,76,77].

Remarkably, depending on the gravity of the lesion, the extent and duration of this physiological disarrangement differ and will eventually carry to multiple organ dysfunction syndrome (MODS). Such condition is caused by systemic inflammatory response syndrome (SIRS) followed transiently by a balancing anti-inflammatory response syndrome (CARS) along with adaptive immunity downregulation [78,79,80].

The pathological mechanisms involve a carefully orchestrated and repeatable genomic storm which constitutes a transcriptional adjustment to acute stress, independently of its provenance [81].

In seriously diseased post-injured patients, this response, along with the high release of PAMPs and DAMPs and the bacterial contamination, could activate further pathological pathways. Consequently, an evolutive MODS will follow, presenting with ARDS, coagulopathy, hepatic and kidney failure.

The underneath pathophysiological mechanism determines the main clinical presentation as follows: thrombotic microangiopathies, coagulative disorders and complement signaling overactivation cause disseminated microvascular thrombosis; pathological triggering of the immune system with subsequent development of severe inflammatory state; immune paralysis with secondary infections due to CD4+, CD8+ and lack of dendritic cells.

In consideration of this overview, the fundamental principle, which would have to act as a beacon light of any efficacious treatment after trauma, is to resolve the injury limiting the further freeing of DAMPs. In this perspective, surgery may be appropriate whenever no other convenient opportunities are available, thereby enabling a faster recovery.

Likewise, liver trauma is associated with hepatic cell death. This is partially due to the injury itself, directly or through the ischemic events, and it could be exacerbated by hemostatic procedures and surgery.

The differentiation between apoptosis and necrosis is crucial regarding hepatocytes. The first one is an uncontrolled breakdown of cells secondary to a severe damage, and the second one is a physiologically inducible and finely planned event. Necrosis leads to a massive inflammatory reaction secondary to the release of lysosomal contents and membrane disruption. Apoptosis requires energy, no collateral damage occurs, and it is characterized by plasma membrane blebbing, chromosomal condensation and nuclear DNA fragmentation [82].

The liver parenchyma in humans has the distinctive capacity of reconstituting following any insult. Although injured lobes do not regenerate in the exact identical manner, a hyperplastic response in the residual parenchyma leads to hypertrophy [83] to restore the lost mass. Typically, the liver regains the majority of its mass in about 2 weeks, regaining its function [84,85,86,87].

Liver regeneration was firstly described by Higgins and Anderson (1931) in an experimental animal model [88] in which a two-thirds partial hepatectomy (PHx) was conducted without damaging the remaining lobes. The result was a broadening of remnant lobes to compensate for the missing parenchyma in one week.

From a pathophysiological point of view, the main cells in charge for liver regeneration after surgical resection or any kind of “trauma” are the hepatocytes that proliferate proportionally to the level of damage [89].

After 48–96 h, the other mature cellular populations (biliary and fenestrated endothelial cells, Kupffer cells, and cells of Ito) start to proliferate, following the mitogenic stimuli from the hepatocytes [90,91,92].

Hepatocyte proliferation starts in the periportal areas of the lobules and then proceeds to the pericentral areas by 36 to 48 h [93].

The hepatic matrix changes from high laminin content to fibronectin and collagen types IV and I.

Hepatic progenitor cells (HPCs), which originates from the canals of Hering [94,95,96], seems to support the process of regeneration. They have been described in chronic hepatopathies [97] and play an essential role in acetaminophen-induced injury [98].

The first considered initial factors with the role of stimulating many other transcriptional factors in hepatic reconstitution were Interleukin-6 (IL-6) and tumor necrosis factor-α (TNF-α) [99,100]. Afterwards, other blood-derived mitogens, as hepatocyte growth factor (HGF), were recognized as potential growth factors in hepatic rebuilding [101].

Webber and colleagues (1994) stated that the role of HGF and others grow factors (i.e., transforming growth factor-α (TGF-α) and heparin-binding epidermal growth factor (HB-EGF) does not rely only in their proliferative stimulus, but first and foremost in their power to act as “priming” signals to switch the hepatic cells into a responsive condition [102]. They recognized signals implicated in the activation of the reaction to damage permitting capable hepatocytes to proceed into their cell cycle.

In addition, this pathway is regulated by co-mitogens (insulin, glucagon, steroid hormones, notably estradiol, and epinephrine) which downregulate growth factor inhibitors (activin A and TGF-β) and promote mitogens function.

The endotoxin lipopolysaccharide (LPS) is one of the few factors that have a primary role in the release of cytokines and growth factors implicated in hepatic reconstitution. Such molecule is a product of Gram-negative microbes of the intestine that acts as a powerful message for Kupffer cells in initiating the process of regeneration.

Other humoral factors that trigger the concerted regenerative response in hepatocytes seem fundamental. Among them, there are the urokinase plasminogen activator (uPA) and its downstream effector plasminogen that cleave the pro-HGF and the extracellular adenosine triphosphate (ATP), determining a fast temporary triggering of c-Jun–amino-terminal kinase (JNK) pathway, induction of early genes FOS and JUN, and AP-1 DNA-binding activity [103,104,105].

This process is counteracted by stimuli to terminate the proliferation when the liver size reaches the functional needs of the organism. In some words, a “hepatostat” might exist as a major controller of the liver/body-mass ratio.

The major inhibitors of hepatic proliferation are TGF-β and associated other components as activin [106]. TGF-β is produced mainly by hepatic stellate cells. In the early phase, it forms inhibitory complexes with SKI proto-oncogene (SKI) and SKI-like proto-oncogene (SnoN) [107], rendering hepatocytes initially resistant to TGF-β [108]. Subsequently, this factor operates through a heteromeric receptor complex, which then phosphorylate proteins of the SMAD family (protein homologs of both the Drosophila protein mothers against decapentaplegic (MAD) and the Caenorhabditis elegans protein SMA), notably SMAD2 and SMAD3 [109].

Reactive oxygen species (ROS) promote synthesis and activation of TGF-β [110], explaining the decreased replication following ischemia and reperfusion.

Many factors may impact the liver’s ability to regenerate: age of the patients and of the liver [111,112]; biliary obstruction or the presence of external biliary drainage for obstructive jaundice impairing enterohepatic circulation may reduce liver regeneration [113]; diabetes mellitus [114,115,116,117]; nutritional status; hepatic diseases [118,119]; male gender [120]; pharmacologic therapy, including frequently prescribed drugs and chemotherapy.

However, the regenerated parenchyma is deformed with an important change in anatomical borders, frequently characterized by a rotation of the portal triad components [121,122,123].

## 13. Conclusions

In conclusion, NOM of liver injury may always be considered in hemodynamically stable patients for every grade of the lesions.

Many operative approaches are helpful to stop the bleeding and to resolve biliary complications of trauma, avoiding or delaying strong surgery, at least until local and systemic inflammatory responses are nearly resolved. 

This attitude may limit acute systemic evolving and chronic hepatic consequences of trauma.

To this end, a flawlessly orchestrated action of the multidisciplinary team is essential to promptly achieve the right decision, avoiding any delay in diagnosis, minimizing mortality and morbidity, and shortening hospital stay.

## Data Availability

Not applicable.

## References

[1] Coccolini F., Panel T.W.E., Coimbra R., Ordonez C., Kluger Y., Vega F., Moore E.E., Biffl W., Peitzman A., Horer T. (2020). Liver trauma: WSES 2020 guidelines. World J. Emerg. Surg..

[2] Fodor M., Primavesi F., Morell-Hofert D., Haselbacher M., Braunwarth E., Cardini B., Gassner E., Öfner D., Stättner S. (2018). Non-operative management of blunt hepatic and splenic injuries-practical aspects and value of radiological scoring systems. Eur. Surg..

[3] DuBose J., Inaba K., Teixeira P.G., Pepe A., Dunham M.B., McKenney M. (2007). Selective non-operative management of solid organ injury following abdominal gunshot wounds. Injury.

[4] Kozar R.A., Feliciano D.V., Moore E.E., Moore F.A., Cocanour C.S., West M.A., Davis J.W., McIntyre R.C. (2011). Western Trauma Association/Critical Decisions in Trauma: Operative Management of Adult Blunt Hepatic Trauma. J. Trauma Inj. Infect. Crit. Care.

[5] Zago T.M., Pereira B.M.T., Calderan T.R.A., Godinho M., Nascimento B., Fraga G.P. (2012). Nonoperative management for patients with grade IV blunt hepatic trauma. World J. Emerg. Surg..

[6] Ciraulo D.L., Nikkanen H.E., Palter M., Markowitz S., Gabram S., Cowell V., Luk S., Jacobs L. (1996). Clinical Analysis of the Utility of Repeat Computed Tomographic Scan before Discharge in Blunt Hepatic Injury. J. Trauma Inj. Infect. Crit. Care.

[7] Poletti P.A., Mirvis S.E., Shanmuganathan K., Takada T., Killeen K.L., Perlmutter D., Hahn J., Mermillod B. (2004). Blunt Abdominal Trauma Patients: Can Organ Injury Be Excluded without Performing Computed Tomography?. J. Trauma Inj. Infect. Crit. Care.

[8] Thapar P.M., Ghawat R.M., Dalvi A.N., Rokade M.L., Philip R.M., Warawdekar G.M., Bapat M.R. (2013). Massive Liver Trauma—Multidisciplinary Approach and Minimal Invasive Surgery can Salvage Patients. Indian J. Surg..

[9] Trauma, 8e; AccessSurgery; McGraw Hill Medical. https://accesssurgery.mhmedical.com/content.aspx?bookid=2057&sectionid=156210710.

[10] Piper G.L., Peitzman A.B. (2010). Current Management of Hepatic Trauma. Surg. Clin. N. Am..

[11] Croce M.A., Fabian T.C., Menke P.G., Waddle-Smith L., Minard G., Kudsk K.A., Patton J.H., Schurr M.J., Pritchard F.E. (1995). Nonoperative Management of Blunt Hepatic Trauma Is the Treatment of Choice for Hemodynamically Stable Patients Results of a Prospective Trial. Ann. Surg..

[12] Cimbanassi S., Chiara O., Leppaniemi A., Henry S., Scalea T.M., Shanmuganathan K., Biffl W., Catena F., Ansaloni L., Tugnoli G. (2018). Nonoperative management of abdominal solid-organ injuries following blunt trauma in adults: Results from an International Consensus Conference. J. Trauma Acute Care Surg..

[13] Velmahos G.C., Toutouzas K.G., Radin R., Chan L., Demetriades D. (2006). Nonoperative treatment of blunt injury to solid abdominal organs:: A prospective study. Arch. Surg..

[14] Brillantino A., Iacobellis F., Festa P., Mottola A., Acampora C., Corvino F., Del Giudice S., Lanza M., Armellino M., Niola R. (2019). Non-Operative Management of Blunt Liver Trauma: Safety, Efficacy and Complications of a Standardized Treatment Protocol. Bull. Emerg. Trauma.

[15] Badger S.A., Barclay R., Campbell P., Mole D., Diamond T. (2009). Management of Liver Trauma. World J. Surg..

[16] Park K.B., You D.D., Hong T.H., Heo J.M., Won Y.S. (2015). Comparison between operative versus non-operative management of traumatic liver injury. Korean J. Hepato-Biliary-Pancreatic Surg..

[17] Pachter H.L., Feliciano D.V. (1996). Complex Hepatic Injuries. Surg. Clin. N. Am..

[18] Bala M., Gazalla S.A., Faroja M., Bloom A.I., Zamir G., Rivkind A.I., Almogy G. (2012). Complications of high grade liver injuries: Management and outcomewith focus on bile leaks. Scand. J. Trauma Resusc. Emerg. Med..

[19] D’Amours S.K., Simons R.K., Scudamore C.H., Nagy A.G., Brown D.R. (2001). Major Intrahepatic Bile Duct Injuries Detected after Laparotomy: Selective Nonoperative Management. J. Trauma Inj. Infect. Crit. Care.

[20] Mohr A.M., Lavery R.F., Barone A., Bahramipour P., Magnotti L.J., Osband A.J., Sifri Z., Livingston D.H. (2003). Angiographic Embolization for Liver Injuries: Low Mortality, High Morbidity. J. Trauma Inj. Infect. Crit. Care.

[21] Griffen M., Ochoa J., Boulanger B.R. (2000). A minimally invasive approach to bile peritonitis after blunt liver injury. World J. Emerg. Surg..

[22] Nathan M., Gates J., Ferzoco S.J. (2003). Hepatic Duct Confluence Injury in Blunt Abdominal Trauma: Case Report and Synopsis on Management. Surg. Laparosc. Endosc. Percutaneous Tech..

[23] Boese C.K., Hackl M., Müller L.P., Ruchholtz S., Frink M., Lechler P. (2015). Nonoperative management of blunt hepatic trauma: A systematic review. J. Trauma Acute Care Surg..

[24] De Backer A., Fierens H., De Schepper A., Pelckmans P., Jorens P.G., Vaneerdeweg W. (1998). Diagnosis and nonsurgical management of bile leak complicated by biloma after blunt liver injury: Report of two cases. Eur. Radiol..

[25] Carrillo E.H., Reed D.N., Gordon L., Spain D.A., Richardson J.D. (2001). Delayed laparoscopy facilitates the management of biliary peritonitis in patients with complex liver injuries. Surg. Endosc..

[26] Coccolini F., Montori G., Catena F., Di Saverio S., Biffl W.L., Moore E.E., Peitzman A.B., Rizoli S., Tugnoli G., Sartelli M. (2015). Liver trauma: WSES position paper. World J. Emerg. Surg..

[27] Johnson J.W., Gracias V.H., Gupta R., Guillamondegui O., Reilly P.M., Shapiro M.B., Kauder D.R., Schwab C.W. (2002). Hepatic Angiography in Patients Undergoing Damage Control Laparotomy. J. Trauma Inj. Infect. Crit. Care.

[28] Chatoupis K., Papadopoulou G., Kaskarelis I. (2013). New technology in the management of liver trauma. Ann. Gastroenterol. Q. Publ. Hell. Soc. Gastroenterol..

[29] Ciraulo D.L., Luk S., Palter M., Cowell V., Welch J., Cortes V., Orlando R., Banever T., Jacobs L. (1998). Selective Hepatic Arterial Embolization of Grade IV and V Blunt Hepatic Injuries: An extension of resuscitation in the nonoperative management of traumatic hepatic injuries. J. Trauma Inj. Infect. Crit. Care.

[30] Sirinek K.R. (2000). Diagnosis and Treatment of Intra-Abdominal Abscesses. Surg. Infect..

[31] Men S., Akhan O., Köroğlu M. (2002). Percutaneous drainage of abdominal abcess. Eur. J. Radiol..

[32] Asai N., Ohkuni Y., Yamazaki I., Kaneko N., Aoshima M., Kawamura Y. (2013). Therapeutic impact of CT-guided percutaneous catheter drainage in treatment of deep tissue abscesses. Braz. J. Infect. Dis..

[33] Roberts B.W. (2015). CT-guided Intra-abdominal Abscess Drainage. Radiol. Technol..

[34] Winter K., Greif V., Crispin A., Burgard C., Forbrig R., Liebig T., Trumm C., Stahl R. (2021). CT-Guided Drainage of Fluid Collections Following Liver Resection: Technical and Clinical Outcome of 143 Patients during a 14-Year Period. Diagnostics.

[35] Lubezky N., Konikoff F.M., Rosin D., Carmon E., Kluger Y., Ben-Haim M. (2005). Endoscopic sphincterotomy and temporary internal stenting for bile leaks following complex hepatic trauma. Br. J. Surg..

[36] Tarnasky P.R., Cunningham J.T., Hawes R.H., Hoffman B.J., Uflacker R., Vujic I., Cotton P.B. (1997). Transpapillary stenting of proximal biliary strictures: Does biliary sphincterotomy reduce the risk of postprocedure pancreatitis?. Gastrointest. Endosc..

[37] Oguz S. (2017). The role of percutaneous transhepatic biliary drainage in the management of massive blunt liver trauma: A case report. Turk. J. Trauma Emerg. Surg..

[38] Hollands M.J., Little J.M. (1991). Post-Traumatic Bile Fistulae. J. Trauma: Inj. Infect. Crit. Care.

[39] Lau W.Y., Lai E.C.H., Lau S.H.Y. (2010). Management of bile duct injury after laparoscopic cholecystectomy: A review. ANZ J. Surg..

[40] Yu W.-Y., Li Q.-J., Gong J.-P. (2016). Treatment strategy for hepatic trauma. Chin. J. Traumatol..

[41] Pilgrim C.H.C., Usatoff V. (2006). Role of Laparoscopy in Blunt Liver Trauma. ANZ J. Surg..

[42] Letoublon C., Reche F., Abba J., Arvieux C. (2011). Damage control laparotomy. J. Visc. Surg..

[43] Anderson I.B., Al Saghier M., Kneteman N.M., Bigam D.L. (2004). Liver Trauma: Management of Devascularization Injuries. J. Trauma Inj. Infect. Crit. Care.

[44] Peitzman A.B., Marsh J.W. (2012). Advanced operative techniques in the management of complex liver injury. J. Trauma Acute Care Surg..

[45] Kobayashi L.M., Costantini T.W., Hamel M.G., Dierksheide J.E., Coimbra R. (2016). Abdominal vascular trauma. Trauma Surg. Acute Care Open.

[46] Nunez R.M., Naranjo M.P., Foianini E., Ferrada P., Rincon E., García-Perdomo H.A., Burbano P., Herrera J.P., García A.F., Ordoñez C.A. (2017). A meta-analysis of resuscitative endovascular balloon occlusion of the aorta (REBOA) or open aortic cross-clamping by resuscitative thoracotomy in non-compressible torso hemorrhage patients. World J. Emerg. Surg..

[47] Gruen R.L., Brohi K., Schreiber M., Balogh Z.J., Pitt V., Narayan M., Maier R.V. (2012). Haemorrhage control in severely injured patients. Lancet.

[48] Polanco P., Leon S., Pineda J., Puyana J.C., Ochoa J.B., Alarcon L., Harbrecht B.G., Geller D., Peitzman A.B. (2008). Hepatic Resection in the Management of Complex Injury to the Liver. J. Trauma Inj. Infect. Crit. Care.

[49] Fang J.-F., Chen R.-J., Lin B.-C., Hsu Y.-B., Kao J.-L., Chen A.M.-F. (2000). Blunt Hepatic Injury: Minimal Intervention Is the Policy of Treatment. J. Trauma Inj. Infect. Crit. Care.

[50] Krige J. (2002). Liver fracture and bleeding. Br. J. Surg..

[51] Hatipoĝlu S., Bulbuloglu B., Piskin T., Kayaalp C., Yilmaz S. (2013). Living Donor Liver Transplantation for Alveolar Echinococcus Is a Difficult Procedure. Transplant. Proc..

[52] Delis S.G., Bakoyiannis A., Selvaggi G., Weppler D., Levi D., Tzakis A.G. (2009). Liver transplantation for severe hepatic trauma: Experience from a single center. World J. Gastroenterol..

[53] Ringe B., Pichlmayr R. (2005). Total hepatectomy and liver transplantation: A life-saving procedure in patients with severe hepatic trauma. Br. J. Surg..

[54] Tucker O.N., Marriott P., Rela M., Heaton N. (2008). Emergency liver transplantation following severe liver trauma. Liver Transplant..

[55] Veroux M., Cillo U., Brolese A., Veroux P., Madia C., Fiamingo P., Zanus G., Buffone A., Gringeri E., D’Amico D.F. (2003). Blunt liver injury: From non-operative management to liver transplantation. Injury.

[56] Esposito S. (2001). Immune System and Surgical Site Infection. J. Chemother..

[57] Mannino M., Toro A., Teodoro M., Coccolini F., Sartelli M., Ansaloni L., Catena F., Di Carlo I. (2019). Open conversion for laparoscopically difficult cholecystectomy is still a valid solution with unsolved aspects. World J. Emerg. Surg..

[58] Kelly M.D. (2009). Laparoscopic retrograde (fundus first) cholecystectomy. BMC Surg..

[59] Dip F., Menzo E.L., White K.P., Rosenthal R.J. (2021). Does near-infrared fluorescent cholangiography with indocyanine green reduce bile duct injuries and conversions to open surgery during laparoscopic or robotic cholecystectomy?—A meta-analysis. Surgery.

[60] Strasberg S.M., Pucci M.J., Brunt L.M., Deziel D.J. (2016). Subtotal Cholecystectomy—“Fenestrating” vs “Reconstituting” Subtypes and the Prevention of Bile Duct Injury: Definition of the Optimal Procedure in Difficult Operative Conditions. J. Am. Coll. Surg..

[61] Low S.-W., Iyer S.G., Chang S.K.-Y., Mak K.S.W., Lee V.T.W., Madhavan K. (2009). Laparoscopic cholecystectomy for acute cholecystitis: Safe implementation of successful strategies to reduce conversion rates. Surg. Endosc..

[62] Kim H.C., Yang D.M., Kim S.W., Park S.J. (2014). Gastrointestinal tract perforation: Evaluation of MDCT according to perforation site and elapsed time. Eur. Radiol..

[63] Siow S.L., Mahendran H.A., Wong C.M., Hardin M., Luk T.L. (2018). Laparoscopic versus open repair of perforated peptic ulcer: Improving outcomes utilizing a standardized technique. Asian J. Surg..

[64] Kumar P., Khan H.M., Hasanrabba S. (2014). Treatment of perforated giant gastric ulcer in an emergency setting. World J. Gastrointest. Surg..

[65] Salminen P., Tuominen R., Paajanen H., Rautio T., Nordström P., Aarnio M., Rantanen T., Hurme S., Mecklin J.-P., Sand J. (2018). Five-Year Follow-up of Antibiotic Therapy for Uncomplicated Acute Appendicitis in the APPAC Randomized Clinical Trial. JAMA.

[66] Yu M.-C., Feng Y.-J., Wang W., Fan W., Cheng H.-T., Xu J. (2017). Is laparoscopic appendectomy feasible for complicated appendicitis? A systematic review and meta-analysis. Int. J. Surg..

[67] Buchweitz O., Malik E., Kressin P., Meyhoefer-Malik A., Diedrich K. (2000). Laparoscopic management of tubo-ovarian abscesses. Surg. Endosc..

[68] Lundorff P. (1997). Laparoscopic surgery in ectopic pregnancy. Acta obstetricia et gynecologica Scandinavica. Supplement.

[69] Aulestia S.N., Cantele H., Leyba J.L., Navarrete M., Llopla S.N. (2003). Laparoscopic diagnosis and treatment in gynecologic emergencies. JSLS J. Soc. Laparoendosc. Surg..

[70] Sozen I., Kadako R., Fleischman S., Arici A. (2001). Diagnosis and laparoscopic management of a fallopian tube torsion following Irving tubal sterilization: A case report. Surg. Endosc..

[71] Cohen S.B., Oelsner G., Seidman D.S., Admon D., Mashiach S., Goldenberg M. (1999). Laparoscopic detorsion allows sparing of the twisted ischemic adnexa. J. Am. Assoc. Gynecol. Laparoscopists.

[72] Bafort C., Beebeejaun Y., Tomassetti C., Bosteels J., Duffy J.M. (2020). Laparoscopic surgery for endometriosis. Cochrane Database Syst. Rev..

[73] Gaitán H.G., Reveiz L., Farquhar C., Elias V.M., Farquhar C. (2014). Laparoscopy for the management of acute lower abdominal pain in women of childbearing age. Cochrane Database Syst. Rev..

[74] Agresta F., Mazzarolo G., Ciardo L.F., Bedin N. (2008). The laparoscopic approach in abdominal emergencies: Has the attitude changed?. Surg. Endosc..

[75] Champault G., Descottes B., Dulucq J.-L., Fabre J.-M., Fourtanier G., Gayet B., Johanet H., Samama G. (2006). Laparoscopie surgery: Guidelines of specialized societies in 2006, SFCL-SFCE. Ann. Chir..

[76] Domínguez L.C., Sanabria A., Vega V., Osorio C. (2010). Early laparoscopy for the evaluation of nonspecific abdominal pain: A critical appraisal of the evidence. Surg. Endosc..

[77] Hori Y SAGES Guidelines Committee (2008). Diagnostic laparoscopy guidelines: This guideline was prepared by the SAGES Guidelines Committee and reviewed and approved by the Board of Governors of the Society of American Gastrointestinal and Endoscopic Surgeons (SAGES), November 2007. Surg. Endosc..

[78] Lund H., Tønnesen H., Tønnesen M.H., Olsen O. (2006). Long-term recurrence and death rates after acute pancreatitis. Scand. J. Gastroenterol..

[79] Leppäniemi A., Tolonen M., Tarasconi A., Segovia-Lohse H., Gamberini E., Kirkpatrick A.W., Ball C.G., Parry N., Sartelli M., Wolbrink D. (2020). Executive summary: WSES Guidelines for the management of severe acute pancreatitis. J. Trauma Acute Care Surg..

[80] Aboulian A., Chan T., Yaghoubian A., Kaji A.H., Putnam B., Neville A., Stabile B.E., de Virgilio C. (2010). Early cholecystectomy safely decreases hospital stay in patients with mild gallstone pancreatitis: A randomized prospective study. Ann. Surg..

[81] Sinha R. (2008). Early laparoscopic cholecystectomy in acute biliary pancreatitis: The optimal choice?. HPB.

[82] Steller H. (1995). Mechanisms and Genes of Cellular Suicide. Science.

[83] Lory J., Schweizer W., Blumgart L.H., Zimmermann A. (1994). The pathology of the atrophy/hypertrophy complex (AHC) of the liver. A light microscopic and immunohistochemical study. Histol. Histopathol..

[84] Kadry Z., Selzner N., Selzner M., Clavien P.-A. (2004). Liver regeneration after adult living donor and deceased donor split-liver transplants. Liver Transplant..

[85] Kamel I.R., Erbay N., Warmbrand G., Kruskal J.B., Pomfret E.A., Raptopoulos V. (2003). Liver regeneration after living adult right lobe transplantation. Gastrointest. Radiol..

[86] Marcos A., Fisher R.A., Ham J.M., Shiffman M.L., Sanyal A.J., Luketic V.A.C., Sterling R.K., Fulcher A.S., Posner M.P. (2000). Liver Regeneration and Function in Donor and Recipient after Right Lobe Adult to Adult Living Donor Liver Transplantation12. Transplant..

[87] Pomfret E.A., Pomposelli J.J., Gordon F.D., Erbay N., Price L.L., Lewis W.D., Jenkins R.L. (2003). Liver Regeneration and Surgical Outcome in Donors of Right-Lobe Liver Grafts. Transplantation.

[88] Higgins G.M., Anderson R.E., Higgins G.M., Anderson R.M. (1931). Experimental pathology of liver: Restoration of liver in white rat following partial surgical removal. AMA Arch. Pathol..

[89] Bucher N.L., Swaffield M.N. (1964). The Rate of Incorporation of Labeled Thymidine into the deoxyribonucleic acid of Regenerating Rat Liver in Relation to The Amount of Liver Excised. Cancer Res..

[90] Gressner A.M. (1995). Cytokines and cellular crosstalk involved in the activation of fat-storing cells. J. Hepatol..

[91] Grisham J.W. (1962). A morphologic study of deoxyribonucleic acid synthesis and cell proliferation in regenerating rat liver; autoradiography with thymidine-H3. Cancer Res..

[92] Widmann J.J., Fahimi H.D. (1975). Proliferation of mononuclear phagocytes (Kupffer cells) and endothelial cells in regenerating rat liver. A light and electron microscopic cytochemical study. Am. J. Pathol..

[93] Mrabes H., Wirsching R., Tuczek H.-V., Iseler G. (1976). Analysis of Cell Cycle Compartments of Hepatocytes after Partial Hepatectomy. Cell Prolif..

[94] Carpentier R., Suñer R.E., Van Hul N., Kopp J.L., Beaudry J., Cordi S., Antoniou A., Raynaud P., Lepreux S., Jacquemin P. (2011). Embryonic Ductal Plate Cells Give Rise to Cholangiocytes, Periportal Hepatocytes, and Adult Liver Progenitor Cells. Gastroenterology.

[95] Carpino G., Cardinale V., Onori P., Franchitto A., Berloco P.B., Rossi M., Wang Y., Semeraro R., Anceschi M., Brunelli R. (2012). Biliary tree stem/progenitor cells in glands of extrahepatic and intraheptic bile ducts: An anatomical in situ study yielding evidence of maturational lineages. J. Anat..

[96] Rodrigo-Torres D., Affò S., Coll M., Morales-Ibanez O., Millán C., Blaya D., Alvarez-Guaita A., Rentero C., Lozano J.J., Maestro M.A. (2014). The biliary epithelium gives rise to liver progenitor cells. Hepatology.

[97] Roskams T., De Vos R., Van Eyken P., Myazaki H., Van Damme B., Desmet V. (1998). Hepatic OV-6 expression in human liver disease and rat experiments: Evidence for hepatic progenitor cells in man. J. Hepatol..

[98] Kofman A.V., Morgan G., Kirschenbaum A., Osbeck J., Hussain M., Swenson S., Theise N.D. (2005). Dose- and time-dependent oval cell reaction in acetaminophen-induced murine liver injury. Hepatology.

[99] Cressman D.E., Diamond R.H., Taub R. (1995). Rapid activation of the Stat3 transcription complex in liver regeneration. Hepatology.

[100] Yamada Y., Kirillova I., Peschon J.J., Fausto N. (1997). Initiation of liver growth by tumor necrosis factor: Deficient liver regeneration in mice lacking type I tumor necrosis factor receptor. Proc. Natl. Acad. Sci. USA.

[101] Fausto N. (2000). Liver regeneration. J. Hepatol..

[102] Webber E.M., Godowski P.J., Fausto N. (1994). In vivo response of hepatocytes to growth factors requires an initial priming stimulus. Hepatology.

[103] Currier A.R., Sabla G., Locaputo S., Melin-Aldana H., Degen J.L., Bezerra J.A. (2003). Plasminogen directs the pleiotropic effects of uPA in liver injury and repair. Am. J. Physiol. Liver Physiol..

[104] Gonzales E., Julien B., Serrière-Lanneau V., Nicou A., Doignon I., Lagoudakis L., Garcin I., Azoulay D., Duclos-Vallée J.-C., Castaing D. (2010). ATP release after partial hepatectomy regulates liver regeneration in the rat. J. Hepatol..

[105] Tackett B.C., Sundararajah T., Mei Y., Maynard J.P., Cheruvu S., Mani A., Hernandez-Garcia A., Vigneswaran N., Karpen S.J., Thevananther S. (2014). P2Y2 purinergic receptor activation is essential for efficient hepatocyte proliferation in response to partial hepatectomy. Am. J. Physiol. Liver Physiol..

[106] Derynck R., Zhang Y.E. (2003). Smad-dependent and Smad-independent pathways in TGF-β family signalling. Nature.

[107] Macías-Silva M., Li W., Leu J.I., Crissey M.A.S., Taub R. (2002). Up-regulated Transcriptional Repressors SnoN and Ski Bind Smad Proteins to Antagonize Transforming Growth Factor-beta; Signals during Liver Regeneration. J. Biol. Chem..

[108] Koniaris L.G., McKillop I.H., Schwartz S.I., Zimmers T. (2003). Liver regeneration. J. Am. Coll. Surg..

[109] Lönn P., Morén A., Raja E., Dahl M., Moustakas A. (2008). Regulating the stability of TGFbeta; receptors and Smads. Cell Res..

[110] Koli K., Myllärniemi M., Keski-Oja J., Kinnula V.L. (2008). Transforming Growth Factor-beta;Activation in the Lung: Focus on Fibrosis and Reactive Oxygen Species. Antioxidants Redox Signal..

[111] Burroughs A.K., Sabin C.A., Rolles K., Delvart V., Karam V., Buckels J., O’Grady J.G., Castaing D., Klempnauer J., Jamieson N. (2006). 3-month and 12-month mortality after first liver transplant in adults in Europe: Predictive models for outcome. Lancet.

[112] Feng S., Goodrich N., Bragg-Gresham J., Dykstra D., Punch J., DebRoy M., Greenstein S., Merion R. (2018). Characteristics Associated with Liver Graft Failure: The Concept of a Donor Risk Index. Am. J. Transplant..

[113] Suzuki H., Iyomasa S., Nimura Y., Yoshida S. (1994). Internal biliary drainage, unlike external drainage, does not suppress the regeneration of cholestatic rat liver after partial hepatectomy. Hepatology.

[114] Starzl T.E., Porter K.A., Kashiwagi N., Putnam C.W. (1975). Portal hepatotrophic factors, diabetes mellitus and acute liver atrophy, hypertrophy and regeneration. Surg. Gynecol. Obstet..

[115] Demori I., Balocco S., Voci A., Fugassa E. (2000). Increased insulin-like growth factor binding protein-4 expression after partial hepatectomy in the rat. Am. J. Physiol. Liver Physiol..

[116] Ghahary A., Minuk G.Y., Luo J., Gauthier T., Murphy L.J. (1992). Effects of partial hepatectomy on hepatic insulinlike growth factor binding protein-1 expression. Hepatology.

[117] Yamada T., Yamamoto M., Ozawa K., Honjo I. (1977). Insulin Requirements for Hepatic Regeneration Following Hepatectomy. Ann. Surg..

[118] Kim T.-H., Bowen W.C., Stolz D.B., Runge D., Mars W.M., Michalopoulos G.K. (1998). Differential Expression and Distribution of Focal Adhesion and Cell Adhesion Molecules in Rat Hepatocyte Differentiation. Exp. Cell Res..

[119] Poynard T., Bedossa P., Opolon P. (1997). Natural history of liver fibrosis progression in patients with chronic hepatitis C. Lancet.

[120] Yokoyama Y., Nagino M., Nimura Y. (2007). Which Gender is Better Positioned in the Process of Liver Surgery? Male or Female?. Surg. Today.

[121] Ikeda K., Kinoshita H., Hirohashi K., Kubo S., Kaneda K. (1995). The Ultrastucture, Kinetics and Intralobular Distribution of Apoptotic Hepatocytes after Portal Branch Ligation with Special Reference to Their Relationship to Necrotic Hepatocytes. Arch. Histol. Cytol..

[122] Shibayama Y., Hashimoto K., Nakata K. (1991). Recovery from hepatic necrosis following acute portal vein embolism with special reference to reconstruction of occluded vessels. J. Pathol..

[123] Goffette P.P., Laterre P.-F. (2002). Traumatic injuries: Imaging and intervention in post-traumatic complications (delayed intervention). Eur. Radiol..

